# Colubrid Venom Composition: An -Omics Perspective

**DOI:** 10.3390/toxins8080230

**Published:** 2016-07-23

**Authors:** Inácio L. M. Junqueira-de-Azevedo, Pollyanna F. Campos, Ana T. C. Ching, Stephen P. Mackessy

**Affiliations:** 1 Laboratório Especial de Toxinologia Aplicada, Center of Toxins, Immune-Response and Cell Signaling (CeTICS), Instituto Butantan, São Paulo 05503-900, Brazil; pollyanna.fc@gmail.com; 2 Laboratório de Imunoquímica, Instituto Butantan, São Paulo 05503-900, Brazil; anatung@gmail.com; 3 School of Biological Sciences, University of Northern Colorado, Greeley, CO 80639-0017, USA; stephen.mackessy@unco.edu

**Keywords:** Colubridae, evolution, proteins, snake, toxins, transcriptomics

## Abstract

Snake venoms have been subjected to increasingly sensitive analyses for well over 100 years, but most research has been restricted to front-fanged snakes, which actually represent a relatively small proportion of extant species of advanced snakes. Because rear-fanged snakes are a diverse and distinct radiation of the advanced snakes, understanding venom composition among “colubrids” is critical to understanding the evolution of venom among snakes. Here we review the state of knowledge concerning rear-fanged snake venom composition, emphasizing those toxins for which protein or transcript sequences are available. We have also added new transcriptome-based data on venoms of three species of rear-fanged snakes. Based on this compilation, it is apparent that several components, including cysteine-rich secretory proteins (CRiSPs), C-type lectins (CTLs), CTLs-like proteins and snake venom metalloproteinases (SVMPs), are broadly distributed among “colubrid” venoms, while others, notably three-finger toxins (3FTxs), appear nearly restricted to the Colubridae (sensu stricto). Some putative new toxins, such as snake venom matrix metalloproteinases, are in fact present in several colubrid venoms, while others are only transcribed, at lower levels. This work provides insights into the evolution of these toxin classes, but because only a small number of species have been explored, generalizations are still rather limited. It is likely that new venom protein families await discovery, particularly among those species with highly specialized diets.

## 1. Introduction

More than one hundred years of biochemical and pharmacological studies have resulted in an exceptional depth of knowledge about snake venoms. The major toxins of the most medically important taxa of venomous snakes were determined by first generation approaches including protein chemistry, comparative pharmacology and cladistics methods borrowed from evolutionary biology. Advances in molecular biology, particularly protein and nucleic acid sequencing techniques, greatly expanded our understanding of compositional complexity, and more recent development in proteomics and early genomics greatly accelerated the pace of cataloguing venoms in exquisite detail. Recent next generation methods, including deep sequencing transcriptomics (RNAseq), genomic sequencing and high resolution mass spectrometry, including top-down proteomics, generally called venomics; (cf. [1,2,3]), have further accelerated the pace of sequence acquisition and compositional analysis and constituted the basis of large-scale biotechnological explorative initiatives (e.g., [4]). These studies collectively created very complete inventories of the toxin families and superfamilies present in species representing significant risk to human health, further refined by a growing knowledge of the relative abundances, post-translational modifications and also structural conservation of proteins across numerous genera [5,6,7,8,9,10,11,12,13,14,15,16,17,18]. As a side product of the accumulation of this knowledge, the observed differences in venom composition among related taxa are becoming appreciated as a productive model for making evolutionary inferences about diversifying selection. In turn, the association of quantified toxins with empirically demonstrated activities have allowed predictions of functional and ecological roles for the species that produce these toxins (e.g., [19,20,21,22]).

However, because most of these efforts were driven by anthropocentric interests in understanding the mechanisms of actions of toxins causing severely debilitating effects, the major focus has been on the species of medical relevance, which are confined to only three families of modern snakes (Viperidae, Elapidae and Atractaspididae). As a consequence, a large part of the biodiversity of venom-producing snakes was not systematically evaluated in the same way, leaving a gap in our knowledge of the repertoire of toxins from other groups of venomous snakes, specifically those that do not typically result in serious human envenomations [23].

The advanced snakes (Caenophidea, superfamily Colubroidea) include a diverse assemblage of species with an evolutionary history of over 100 million years, most of which possess a venom production system [24,25,26,27]. The family “Colubridae” formerly referred to any caenophidian snake not included in the three medically important families of venomous snakes, and this assemblage, though acknowledged to be a paraphyletic group, resisted systematic consensus for many years [28,29,30,31]. More recently, several groups have reclassified the “Colubridae” into several families and subfamilies [32,33,34,35], but a true consensus classification is still lacking. In a more formal definition, a “colubrid” snake refers only to species belonging to the family Colubridae, which currently includes the subfamilies Natricinae, Pseudoxenodontinae, Dipsadinae, Scaphiodontophiinae, Calamariinae, Grayiinae and Colubrinae [34]. This family represents about 50% of the extant snake fauna (distributed in more than 1800 species), many with very distinct habits and diversification of species within each subfamily, and this classification scheme likely still masks considerable differentiation. Additional rear-fanged species, accounting for approximately 361 species, have been allocated to the families Homalopsidae and Lamprophiidae [34]; further, some authors consider Natricidae (approximately 226 species) and Dipsadidae (approximately 754 species) as distinct families [36,37]. Hereafter, we will use a broader definition of the term “colubrid” to refer to any of the families in the above paraphyletic group and not only to the family Colubridae sensu stricto, though the vast majority of data discussed here come from this family.

In spite of the uncertainties in phylogenies, snakes in these families often possess one or more enlarged rear teeth (opisthoglyph dentition) that are typically associated with a pair of Duvernoy’s venom glands, homologs of the venom glands of families Viperidae, Elapidae and Atractaspididae [38,39]. Several species show no specialized or enlarged rear fangs (aglyph dentition), though in some cases they also contain other specialized oral glands that produce venom-like secretions [40]. 

Colubrids are rarely investigated using -omics approaches mainly because of their limited capacity to inject a debilitating dose of venom into humans, and so the biological activity of most species’ venoms is wholly unknown. As are all snakes, they are predators, and venom is presumed to be of critical importance for capturing, killing and/or digesting the prey [41]. Thus, their venoms are expected to be highly efficient within the proper ecological context of each species, meaning that their venoms could be as rich and diverse in protein types as those from medically important species. Moreover, because the different colubrid venoms are utilized in very distinct ecological scenarios and evolved under different selective pressures, they may contain cryptic novel and unpredictable types of proteins.

Because so few studies have focused on colubrids venoms and a plethora of different methodological approaches were used by different labs, it is not clear which types of toxins are currently known in the various groups, what structural characteristics are known and what their evolutionary history has been. Some of the toxin sequences were obtained through direct protein purification/sequencing, while others were deduced from transcriptomic and/or proteomic investigations. In addition to the different times when the investigations were performed, their specific goals sometimes hindered the perception of unusual new toxins. As a consequence, this has produced a distorted view of toxin repertoires that exist in colubrid venoms and hinders a more complete reconstruction of the evolutionary history of venom protein classes. The somewhat myopic view of venoms as occurring only in front-fanged snake species has interfered with a more holistic, fundamental perspective of the processes underpinning the evolution of venom, restricting the use of these exceptionally diversified animals as models for testing adaptive evolution by natural selection and negatively impacting the discovery of new bioactive molecules. 

Here we survey previously discovered and several new venom proteins from venoms of colubrid species, focusing on those with known protein or cDNA-based sequences. Our intent is to provide an up-to-date catalog of proteins known to occur in colubrid snake venoms and present these in an evolutionary context, highlighting their (presently known) diversification. There are well over 2200 species of non-front-fanged snakes, many of which possess a Duvernoy’s venom gland, so it should be immediately apparent that there is much work to do before a well-documented understanding of venom diversity among colubrids is possible. Nonetheless, by summarizing known information, we hope that this report will stimulate further investigation of the many genera of colubrid snakes for which we have no toxinological information.

## 2. Results and Discussion

### 2.1. Compiling the Venom Components of Colubrid Snakes

**Figure 1:**
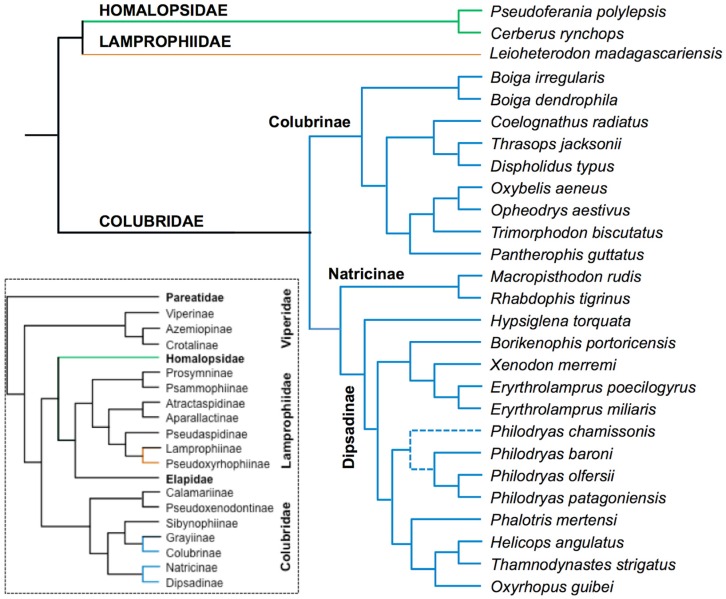
Schematic cladograms showing the phylogenetic relationships among families and species of snakes discussed in this work (colored branches). The cladogram was based on the phylogenetic tree proposed by Pyron et al. [34]. Dashed lines in *Philodryas* indicate the presumed placement of *P. chamissonis*.

Our attempt to compile the toxins present in colubrids was based on three strategies: (1) generating transcriptomic sequences from the venom glands of three species of colubrids, *Erythrolamprus miliaris*, *Oxyrhopus guibei* and *Xenodon merremi* (Dipsadinae subfamily of Colubridae), to identify transcripts coding for known and putative types of snake toxins (Table S1); (2) prospecting public databases for toxin-related sequences in other colubrid species previously investigated; and (3) reviewing the literature on colubrid venoms that describes the isolation of toxins or provides clear evidence for the occurrence of specific proteins in colubrid venoms. For ease of presentation, the protein types compiled were organized into three categories: (a) “major snake venom components” (Table 1), referring to protein types generally encountered in high amounts in the venoms of many species of traditionally venomous snakes (Viperidae, Elapidae and Atractaspididae) and which certainly are important toxins; (b) “minor (or arguably) venom components” (Table 2), referring to protein types previously described in the venom of some species of venomous snakes, generally in low amounts, and which may represent toxins, ancillary venom proteins or housekeeping proteins; and (c) “putative new snake toxins in colubrid venoms” (Table 3), referring to protein types uncovered from colubrid venom analyses, occurring in high or low quantities, which may represent putative toxins, exclusive or not to the group. We should emphasize that the separation into major and minor components is unrelated to the level of expression (or protein quantity) of the components in colubrid venoms. Rather, it is related to a relative importance and frequency of the proteins in venoms of other venomous snakes. This organization is admittedly subjective and flexible, but it was adopted because it would be unrealistic to propose a division based on more tangible (but highly diverse) measures provided by the varied methodologies adopted in the studies reviewed. Because it reflects a particular point of view, it does not aim to establish a strict rule for toxin categorization or to define whether certain venom proteins do or do not have relevant functions in snake venoms. Additionally, because the strict definition of “toxin” would be dependent on the functional, ecological and behavioral contexts of the species, which are largely unavailable for colubrids, the protein types included here should be generally regarded as “venom components”, which in some cases are very likely to be toxins and in other cases may or may not be toxins. The approximate phylogenetic relationships among the species for which venom components could be identified in our compilation are depicted in trees (Figure 1) based on the phylogenetic hypothesis of Colubroidea snakes as proposed by Pyron et al. [34]. 

It is interesting to note that most -omics characterizations of colubrid venoms have addressed members of the Dipsadinae subfamily of Colubridae, perhaps because a large number of genera in this subfamily are rear-fanged and possess Duvernoy’s venom glands, and several have been involved in human envenomations, typically with mild effects [63,64,65]. The Dipsadinae species studied include *Philodryas olfersii* [59], *Thamnodynastes strigatus* [60] and *Hypsiglena* sp. [12], as well as *Erythrolamprus miliaris*, *Oxyrhopus guibei* and Xenodon merremi described here. For Colubrinae, transcriptomes of oral glands from *Pantherophis guttatus, Opheodrys aestivus* [9] and *Boiga irregularis* (Duvernoy’s venom gland); [12] were generated, although only the last one was complemented by venom proteomic analysis. Nevertheless, many toxins from the other subfamilies have been investigated by more focused approaches, such as protein purification from the venom (e.g., *Borikenophis portoricensis* [47]) or specific cDNA cloning, including some genera with particularly toxic venom, such as the natricine *Rhabdophis* [66]. Very recently, full length mRNAs derived from secreted venoms of several colubrine and dipsadine colubrids were reverse transcribed and sequenced, demonstrating that it is possible to obtain transcript sequences from venom alone [67].

### 2.2. Major Snake Venom Enzymatic Components

**Figure 2:**
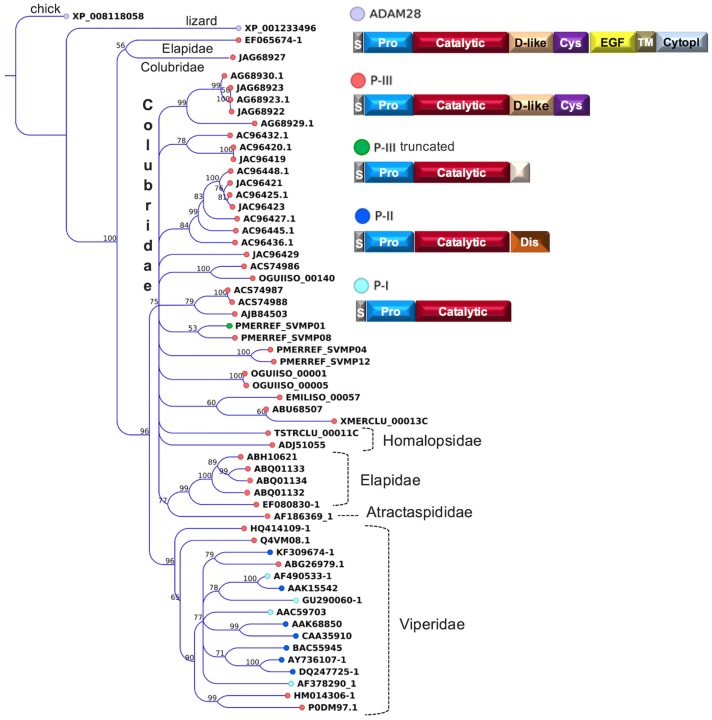
Maximum likelihood tree showing the relationship among representative SVMPs from different snake families. Bootstrap values are plotted close to the internal nodes. Colors in the terminal nodes indicate the types of the precursors, and their domain arrangements are depicted on the right. Abbreviated domains are: S, signal peptide; Pro, prodomain; Catalytic, metalloproteinase; D-like, disintegrin-like; Dis, disintegrin; Cys, cysteine rich; TM, transmembrane; EGF, epidermal growth factor; and Cytopl, cytoplasmic. The protein sequences are referred to by their accession numbers in GenBank, except those initiated by the codes EMILISO, OGUIISO, PMERREF, TSTRCLU and XMERCLU, which are mentioned in the “definition” field of sequence files deposited in the Transcriptome Shotgun Assembly (TSA) database.

For most colubrid species, especially in the subfamily Dipsadinae, snake venom metalloproteinases (SVMPs) are predominant components in the transcriptomes and in the proteomes. All sequences described in Colubridae to date belong to the P-III class of SVMPs, which include pre- and pro-domains, a metalloproteinase catalytic domain, a disintegrin-like domain and a cysteine-rich domain (Figure 2). The absence of P-II, P-I and short coding disintegrins in colubrid venoms is in accordance with the hypothesis that those proteins evolved within the family Viperidae from a P-III ancestor gene, after the split of this lineage [68,69]. A solely exception in Colubridae is the occurrence of a shortened P-III SVMP in *Phalotris mertensi*. This protein was proteomically confirmed in the venom of the species and it has a partial disintegrin-like domain and no Cys-rich domain, as a result of a transcript with an early stop codon and a substituted 3′UTR sequence [57]. A phylogenetic tree of representative SVMPs indicates that, despite a high degree of diversity among the Colubridae SVMPs, they share a common ancestor with elapid and atractaspidid P-III SVMPs (Figure 2).

Snake venom serine proteinases (SVSPs) are detected in some colubrid venoms and transcriptomes; however, they are not commonly present in these venoms, nor as abundantly expressed and diversified as observed in many viperid snakes. The few colubrid SVSPs sequenced are related to the kallikrein-like enzymes well characterized in viperid venoms, and they include a C-terminal extension that distinguishes them from the lizard venom kallikrein-like enzymes [70]. 

Phospholipases A_2_ (PLA_2_) are very common components in the venoms of the medically important snake families Elapidae and Viperidae, and they belong to type I and II PLA_2_s, respectively. In colubrids, they seem to not be among major components and have been detected in only a few species. In the colubrine *Trimorphodon biscutatus,* an enzyme was purified and its partial sequence indicated that it was a type IA PLA_2_ [61]. However, in another colubrine (*Dispholidus typus*) [51], in the dipsadine *Oxyrhopus guibei* (this work) and in the pseudoxyrhophiine (family Lamprophiidae) *Leioheterodon madagascariensis* [51], among others, the reported type of PLA_2_ is IIE.

The occurrence of transcripts coding for enzymes of IIE subtype in the venom glands indicates a possible independent recruitment of a PLA_2_ to the venom, since they are distinct from the type IIA paralogs commonly expressed in the venom glands of viperid snakes [51]. Whether or not these type IIE PLA_2_s represent truly new toxins or accessory proteins of the venom glands remains to be clarified, but Hargreaves et al. [9] found them to be exclusively expressed at low levels in the venom glands of the species tested. 

Despite being very common in the venom of other groups of snakes, L-Amino Acid Oxidase (LAAO) was thought to be essentially non-existent in colubrid venoms (e.g., [71]). Very low levels of LAAO activity were detected in Brown Treesnake (*B. irregularis*) venom [72]; however, assays of venom from 13 different species of colubrine, dipsadine and natricine rear-fanged snakes detected no LAAO activity [73]. In a comparative transcriptomic analysis of tissues from *Pantherophis guttatus*, an LAAO was shown to be expressed in the scent gland but not in the salivary glands of this species [9], suggesting it is not a venom component. Recently, however, an LAAO was found moderately expressed in the venom glands of the colubrid *Phalotris mertensi*, and the MS/MS spectrometric analysis clearly showed its presence in the venom of this species [57].

### 2.3. Major Snake Venom Non-Enzymatic Components

Three-Finger Toxins (3FTx) are major constituents of Elapidae venoms and represent the lethal component of the majority of species of this family. These toxins seem to have differential importance in different subfamilies of colubrids. Alpha-colubritoxin from *Coelognathus radiatus* was the first colubrid toxin isolated and sequenced [49] and several other 3FTx, such as denmotoxin and irditoxin, functionally characterized in members of the subfamily Colubrinae, were demonstrated to be abundant toxins with taxon-specific activities [42,45]. The -omics characterization of *Boiga irregularis* venom showed that 3FTx dominate the transcriptome of this species (67.5% of toxin transcripts) [12]. The authors described 58 unique 3FTx sequences grouped into at least 10 sequence clusters that were proteomically confirmed in the venom. These clusters could be arranged in three groups based on the structural characteristics, but none of them were closely related to the above-mentioned irditoxin from the same species. Together with individual sequences isolated from other genera [9,50,56], 3FTx seem to be major components in many venoms of the subfamily Colubrinae. In contrast, in the Dipsadinae, 3FTx are not found [59,60] or are detected at minor abundance and diversity levels [57]. However, in the current work, we retrieved sequences from the transcriptome of the Duvernoy’s venom gland of *Xenodon merremi* (a dipsadine colubrid) that were expressed at high level (Table 1). 3FTx-like sequences were also reported in venom glands of the family Lamprophiidae, as well as in species at the base of the Alethinophidia snake radiation (including species in the Cylindrophiidae and Pythonidae [50]). Nevertheless, it appears likely that 3FTx-like transcripts found in gland tissue of these latter two families may represent house-keeping genes, rather than toxins [9,74].

**Figure 3:**
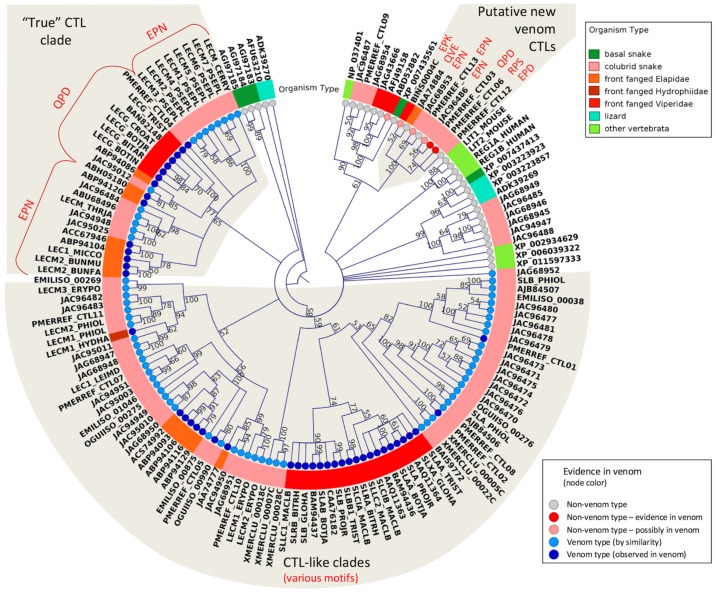
Maximum likelihood circular cladogram showing the relationship among representative CTLs from different snake families. Bootstrap values are plotted close to internal nodes. Colors at the terminal nodes (circles) indicate typical vs. atypical venom proteins and the evidence of occurrence in the venoms. Colors in the diagram surrounding the cladogram indicate the taxonomic groups. The carbohydrate binding motifs, as discussed in the text (EPN, QPD, etc.), are indicated by red type. The protein sequences are referred by their GenBank accession numbers, except those initiated by the codes EMILISO, OGUIISO, PMERREF and XMERCLU, which are mentioned in the “definition” field of sequence files deposited in TSA.

C-Type Lectins (CTL) are ubiquitous venom components in many snake groups, and they are also found abundantly in colubrid venoms. In Colubridae, amounts of venom CTL transcripts vary from 2% in *Philodryas olfersii* [59] to as much as 21% in *Phalotris mertensi* of total transcripts [57]. They were also reported in the snakes *Pseudoferania polylepis* and *Cerberus rynchops* (family Homalopsidae) and were highly expressed in *Cerberus* [48]. From a phylogenetic tree of all colubrid CTLs and related orthologs (Figure 3), it is possible to observe the existence of distinct types of CTLs in non-front fanged snakes, although the phylogenetic reconstruction failed to resolve the evolutionary relationships among them. Nevertheless, in addition to the presence of a CTL-like (snalec) clade, largely found in colubrids, colubrid sequences are observed to be nested within the clade of Elapidae and Viperidae “true” CTLs sequences (i.e., those with a predicted galactose binding motif QPD substituting the plesiotypic motif EPN: [75,76]. One of them (PMERREF_CTL04) was confirmed in *P. mertensi* venom and in fact has the QPD motif, indicating that predicted galactose-binding lectins should also be present in other Colubridae venoms. Moreover, some orphan transcripts observed in the venom glands of snakes from different families clustered completely outside of the clades of typical venom CTLs (Figure 3). Some of them are suggested to code for venom proteins, such as two transcripts highly expressed and proteomically detected in the venom of *P. mertensi*, and similar transcripts are expressed at moderate levels in the venom glands of other colubrids (*Hypsiglena* sp. and *Boiga irregularis*; [12]), viperids (*Bothrops insularis*; [77]) and elapids (*Hoplocephalus bungaroides*; [78]). Interestingly, the encoded proteins from the transcripts of this group present not a single but various motifs (QPD, EPD, EPN, RPS, QVE, and EPK) for sugar binding at the second loop of the carbohydrate recognition domain. It indicates that these genes may have undergone a diversification process that parallels that experienced by other CTL types, i.e., the substitution of the binding motif of the original sugar ligand, mannose, by binding motifs to other types of carbohydrates. 

Although the role of cysteine rich secretory proteins (CRISPs) in venom is not yet clear, they are very ubiquitous venom components and are found in almost all snake species, including colubrids, and have been investigated via either classical protein techniques (e.g., [79]) or -omics profiling [80]. Contrary to the other highly expressed snake toxins, CRISPs seem to have not undergone multiple duplications during snake lineage evolution, and a single paralog is normally found abundantly expressed and translated to a venom protein in each colubrid species; in some species, such as *B. irregularis*, a minor isoform is also present in the venom (Mackessy, unpub. obs.). Nevertheless, positive Darwinian selection on CRISPs were observed to be higher in Colubridae and Viperidae proteins than on other reptiles, while negative selection occurs in mammalian CRISPs [80]. 

**Figure 4:**
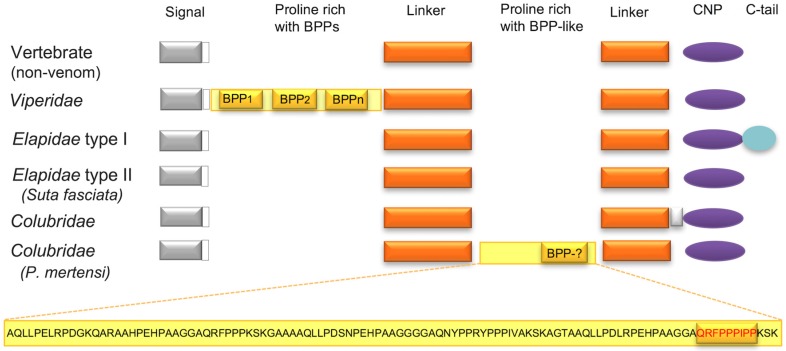
Schematic organization of CNP (and BPP) precursors in the different snake families and in other vertebrates. The precursor of *P. mertensi* [57] exhibits a Pro-rich insertion in the linker region (detached at the bottom), which includes a BPP-like segment that may generate a BPP after processing.

The first C-type natriuretic precursor (CNP) from a colubrid species was described from the *P. olfersii* transcriptome, where it was suggested to have a common ancestor with the natriuretic peptide precursor of elapid snakes and with the bradykinin-potentiating peptides precursor (BPP) of viperid snakes [59]. Currently, nine colubrid species in the three major subfamilies of Colubridae (Colubrinae, Dipsadinae and Natricinae) were shown to have this precursor generally highly expressed in the Duvernoy’s venom glands. Most of them have the same general structure, i.e., the C-type peptide has no C-terminal extension and the CNP prodomain is not preceded by a BPP-containing region (Figure 4). Based on this organization, Jackson et al. [78] suggested that the acquisition of the *C*-terminal extension occurred within the Elapidae, while the acquisition of BBP repeats occurred along the viperid lineage diversification. We notice, however, a notable exception in the CNP precursor of the Dipsadinae *P. mertensi*: this precursor, transcribed at high levels in the venom glands, possesses a long sequence inserted at the middle of the CNP prodomain (linker domain), which is rich in Pro residues (including PP and PPP internal peptides) and resembles the BPP-containing region of the viperid precursor (Figure 4). At the C-terminal portion of this region, one particular motif, “QRFFPPPIPP”, shows a high degree of similarity to the BPP signature. Besides the classical BPPs, which led to the development of successful anti-hypertensive drugs [81], the BPP precursors of Viperidae snakes were demonstrated to generate other bioactive peptides, including SVMP inhibitors [82,83,84,85]. It is thus reasonable to suppose that this region of the *P. mertensi* CNP precursor could also be processed to generate bioactive peptides and perhaps a BPP-like peptide. 

Crotamine is a beta-defensin-type polypeptide very well characterized from rattlesnake (Viperidae) venoms and thought to be restricted to the genus *Crotalus*. However, beta-defensin homologous genes were found in other viperid genera [86] and, more recently, venom gland transcripts were reported at relatively high expression levels in the transcriptome of the colubrids *Thamnodynastes strigatus* [60] and *Phalotris mertensi* [57]. In the latter, the corresponding protein was detected by shotgun MS/MS analysis of the venom, suggesting it may be a valid colubrid venom component. The colubrid proteins have a highly conserved signal peptide, almost identical to that of crotamine (see Supporting Figure 3 from [60]); the mature polypeptides display the same cysteines involved in the disulfide arrangement of crotamine, but the other residues are highly variable, making it difficult to establish the evolutionary relationship between them. 

Kunitz-type proteins appear in snake venoms in several forms, sometimes as single-product precursors (KUN-1), at other times with tandem repeated domains (KUN-2), and less frequently associated with WAP domains in a protein designated ku-wap-fusin (KU-WA-FU) [87]. Although in some species of colubrids these components have a transcriptional level not indicative of a relevant participant in venom, in at least two species, *Hypsiglena* sp. [12] and *Phalotris mertensi* [57], they have medium or elevated expression levels and were also detected in the venom. In *Phalotris mertensi*, three single-domain precursors are highly expressed and dominate the venom profile. The conservation of residues believed to be the protease inhibitory sites in their sequences [88,89] indicate they likely act as serine proteinase inhibitors, the plesiotypic function of this toxin, rather than as neurotoxins, as observed in some elapid Kunitz-like proteins.

### 2.4. Minor or Arguably Actual Venom Components

Other protein components previously reported in the venoms of the families Elapidae and/or Viperidae, generally in minor quantities, are also detected in low amounts in colubrid venoms and/or transcriptomes and are listed in Table 2. However, the actual contribution of these molecules to the venom is debatable, and some authors consider them non-toxins because of their occurrence in non-venom gland specific tissues [9].

Regarding minor occurring enzymes, venom-like acetylcholinesterase (Ache) sequences are found in many elapid species but were suggested as a colubrid venom component only in *Boiga* venom, where low activity was detected [44,73], as well as low expression levels in the transcriptome of *B. irregularis* [12]. A 5′-nucleotidase, on the other hand, was identified at low expression levels in the *Phalotris mertensi* transcriptome, and it was also detected in its venom proteome [57]. Factor Va- and Factor Xa-like proteins are venom serine proteinases distinct from the classical SVSPs [90], and they are believed to have been recruited into the venom proteome on the basis of their occurrences in venoms of the Australian elapid radiation [50]. Accordingly, no Factor Va-like sequence were retrieved in our searches, while only the endophysiological (non-venom) Factor Xa transcripts could be found in the venom glands of three colubrid species but were never identified in the secreted venom. These data indicate that the expression of endophysiological Factor Xa also may occur in the venom glands, in addition to the liver, although at low levels (this work and [9]). Transcripts for some other minor venom enzymes were only found at very low levels. 

Growth factor sequences from colubrids and other snakes, such as vascular endothelial growth factor (VEGF) and nerve growth factor (NGF), are common in the databases. However, there is no clear evidence of them as venom components among colubrids. Whereas a venom-specific VEGF (VEGF-F) was extensively demonstrated to be a venom component in viperid snakes, possibly acting as a toxin dispersion agent [91], an endophysiological paralog (VEGF-A) was later shown to be co-expressed in venom glands and secreted only in low amounts in the venom of some species [92]. All the VEGF forms retrieved from colubrids are similar to VEGF-A, expressed at low levels, and thus they are more likely to be non-toxins, possibly corresponding to the endophysiological factor produced in the venom gland environment. The colubrid NGF sequences available in databases are mostly derived from phylogenetic studies based on this genetic marker. In contrast to other venomous snake families, where NGF was clearly demonstrated to be a venom component, in colubrids there is no support for this factor as a venom component, since it is not specifically transcribed in the venom glands of any species and the protein has to date not been isolated from the venom.

Cobra venom factor (CVF) was clearly demonstrated as a venom component only in elapid snakes [93]. Although very similar sequences could be found expressed at low levels in some colubrids, the absence of protein detection in their venom suggests the transcripts could also be the endophysiological complement factor C3 expressed by blood cells within the venom glands. 

Enzymatic inhibitors that typically function to protect snakes from the bites of other snakes are mainly produced in the liver and secreted into the plasma of venomous and non-venomous snakes [94], but some of them seem to be produced in the venom glands. For example, a specific paralog of a gamma-PLA_2_ inhibitor (gPLA_2_i) was shown to be exclusively expressed in venom glands of *B. jararaca* (Viperidae) [10]. Accordingly, we could identify three colubrid species showing low to medium expression levels of gPLA_2_i, and one of them was proteomically demonstrated in the venom. Protease inhibitors such as cystatins have been previous demonstrated in snake venoms [95], but their role in the venom is unclear. We retrieved transcripts coding for these proteins from some colubrids, but according to the analysis of Hargreaves et al. [9], they have undifferentiated levels of expression among tissues, and no further evidence of their presence in colubrid venoms have been noted yet, indicating that they are probably not colubrid venom components. Nevertheless, the common occurrence of many transcripts coding for all these toxin-like proteins in venom-producing tissues indicate that if they are not toxins, they may play important roles in the maintenance of this specialized secretory epithelium. We did not find transcripts related to sarafotoxins [96] in any colubrids, including *Leioheterodon madagascariensis* (Lamprophiidae), indicating that this component may be apotypic of Atractaspidinae. 

### 2.5. Putative New Snake Toxins Suggested from Colubrid Venoms

**Figure 5:**
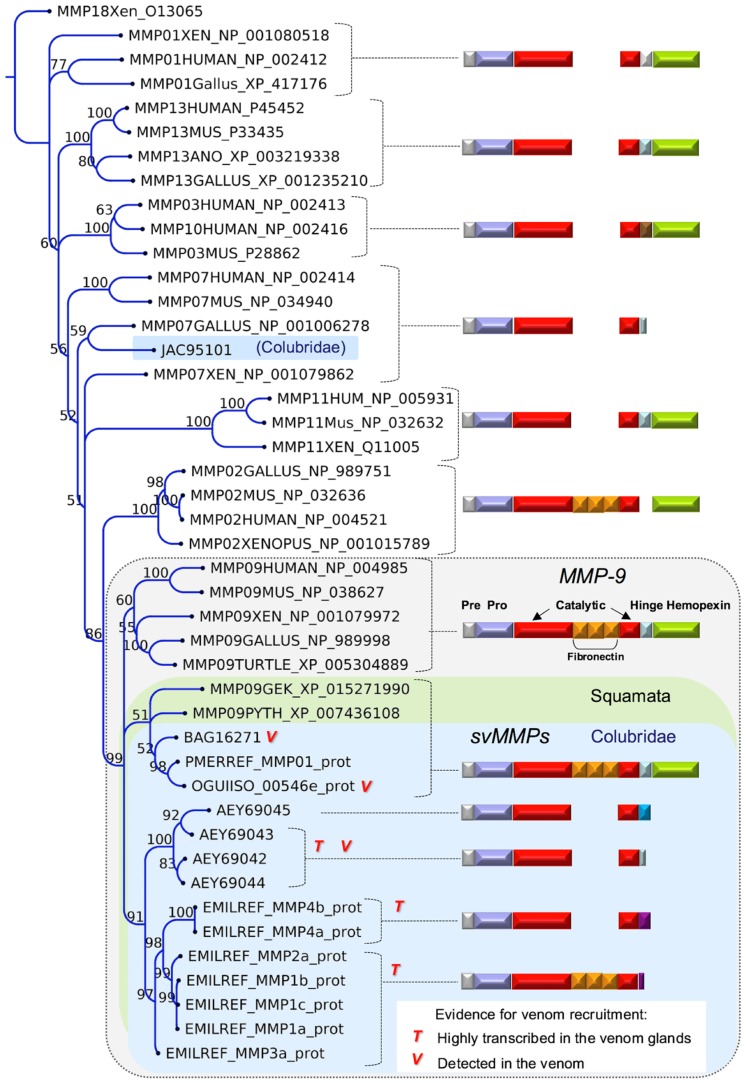
Maximum likelihood tree showing the relationship among svMMPs from different snake families and MMPs from other vertebrate groups. Bootstrap values are plotted close to internal nodes. The domain arrangement of each precursor type is depicted on the right. The types of evidence for the occurrence in venoms are indicated by “T” (transcribed) and “V” (detected in venom). The protein sequences are labeled by their accession numbers in GenBank, except those initiated by the codes EMILREF, OGUIISO, and PMERREF, which are mentioned in the “definition” field of sequence files deposited in TSA.

Although SVMPs dominate many colubrid venom profiles, another type of metzincin, the snake venom matrix metalloproteinase (svMMP), was revealed to be a colubrid-specific venom component likely playing an important role in some species. svMMPs were abundantly found in the transcriptome and proteome of *Thamnodynastes strigatus* [60] and are highly expressed in the transcriptome of *Erythrolamprus miliaris* (this work), both Dipsadinae. Other Dipsadinae species investigated by similar -omics approaches showed lower abundance of svMMPs, though they were still detected in the venoms of some species. A svMMP was also purified, sequenced and functionally characterized from the venom of *Rhabdophis tigrinus* (Natricinae) [66]. For many of the species in which svMMPs were detected, SVMPs were also present in the venom, seemingly indicating that svMMPs are not substituting for the function of SVMPs but perhaps are adding a possible synergistic effect toward producing extracellular matrix lesions caused by the venoms. The colubrid svMMPs show important differences related to the presence of ancillary domains, as illustrated in Figure 5: whereas in some species, such as *Rhabdophis tigrinus*, the protein has a classical MMP9-like structure, in others, such as *Thamnodynastes strigatus*, they do not include the fibronectin repeats nor the hemopexin domains, thus resembling a MMP7-like arrangement. The *E. miliaris* svMMPs found in this work revealed a more complex situation, since some of the precursors have the fibronectin repeats inserted in the catalytic domain, whereas other precursors do not show these domains (Figure 5). Both forms are highly transcribed in the venom glands, representing the major toxin type found in the transcriptome of this species, but unfortunately, we did not have access to the venom of this species to evaluate its effective secretion. It is interesting to observe in a phylogenetic tree of svMMP precursors (Figure 5) that there is a strong clustering of svMMPs within the MMP-9 clade. This result indicates that all svMMPs seem to derive from a single MMP-9 ancestor gene, regardless of the presence or the absence of ancillary domains. Additionally, the clustering of species-specific proteins in monophyletic groups signifies intra-clade gene duplications, with independent losses of the fibronectin and hemopexin domains in some clades (*Thamnodynastes* and *Erythrolamprus*). Moreover, it clearly points out that the simplified MMP7-like arrangement observed in some svMMPs is a derived trait from the modification of a MMP9-type svMMP, rather than originating from an MMP7 gene.

Another enzyme representing an example of a putative toxin from colubrid venom is an acid lipase (svLIPA), similar to mammalian lysosomal acid lipases. In *P. mertensi*, this protein was proteomically and immunochemically detected in the venom and its mRNA was highly expressed in the venom glands [57]. Interestingly, this *P. mertensi* sequence is closely related to acid lipases previously suggested as possible venom components in species of other snake families but not clearly demonstrated in their venoms [97,98], as well as a prominent protein component of saliva from several species of *Varanus* (BLAST search). By comparing acid lipase sequences from different reptiles, we could demonstrate that all transcripts showing evidence for venom proteins in different snakes (i.e., high expression in the venom glands, proteomics detection, or immunoreactivity in venom) form a monophyletic group, and thus LIPA may represent a novel type of venom component, and perhaps a toxin [57].

Novel non-enzymatic components were also proposed from the venoms of non-front fanged snakes. Venom ficolin (veficolin) is a class of putative toxins initially characterized from the homalopsid *Cerberus rynchops* venom and transcriptome [48]. Other related transcripts could be retrieved from several colubrid species but they are generally expressed at low levels, and the encoded proteins were not detected in any other venom. A lactadherin-like protein, a secreted carrier protein containing a FA58C (coagulation factor V and VIII C-terminal) domain, was first identified from a partial clone in the transcriptome of *T. strigatus*. Since it was found proteomically in the venom of that species, it was suggested as a possible venom component [60]. In the present work, we identified a complete transcript coding for this protein in the *Oxyrhopus guibei* transcriptome, but we did not evaluate the venom of this species. A search for similar transcripts in other snakes revealed a complete sequence only in the transcriptome of the viperid *Crotalus horridus* (JAA96713, [15]). An EGF repeat-containing cDNA was found in relatively high levels in the transcriptome of *T. strigatus* but was not confirmed in this venom nor was it retrieved from other species [60]. 

**Figure 6:**
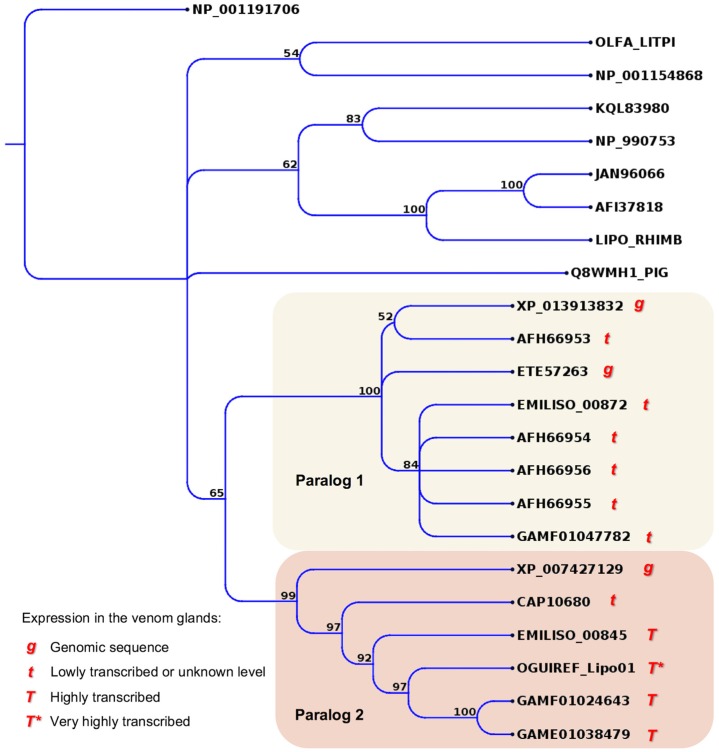
Maximum likelihood tree showing the relationship among lipocalin proteins from different snake families and from other vertebrate groups. Note that transcripts highly expressed in venom glands are all in the same clade. Bootstrap values are plotted close to internal nodes. The protein sequences are labeled by their accession numbers in GenBank, except those initiated by the code EMIL, which is mentioned in the “definition” field of sequence files deposited in TSA and the sequence OGUIREF_Lipo1 (Accession Number KX450875). Sequences labeled GAMF from *A aterrima* were translated from the original nucleotide contigs retrieved from TSA.

An interesting case of a potentially new venom component identified from Colubridae -omics analysis is a type of lipocalin. Transcripts coding for lipocalin-structured proteins were retrieved from several snake venom glands by transcriptomic analysis or by RT-PCR amplification and they were shown to be homologous [51]. In the transcriptomic analysis of the Atractaspidinae *Atractaspis aterrima*, some lipocalin sequences were identified as among the most expressed transcripts in the venom glands [18]. Since lipocalins are common components from some invertebrate venoms and from the saliva of hematophagous animals [99], they were suggested as possible venom components [47]. These proteins also show weak sequence similarities to a putative olfactory protein specifically expressed in high amounts in the Bowman’s glands of the olfactory tissue from a frog [100]. Interestingly, among the original data generated in the present work, we found an extremely highly expressed transcript coding for a lipocalin in *Oxyrhopus guibei*. Alone, this mRNA accounts for 29% of the sequencing reads in the transcriptomic analysis. A phylogenetic tree of all available lipocalin sequences, from snakes and from several other sources, showed that the transcripts highly expressed in snake venom glands, including those from Colubridae and Atractaspidinae, are likely to be orthologs, whereas other transcripts expressed at low levels correspond to a paralogous snake gene (Figure 6). Although it is not possible to confirm, without a proteomic analysis, if lipocalin is indeed a venom component, the high expression of the same gene in the venom glands of distinct snake species suggests that its product should have an important role for this animal, perhaps as a new toxin or perhaps involved in olfactory-mediated behavior.

Finally, a putative new toxin proposed from a highly expressed transcript from *Atractaspsis aterrima* (Atractaspidine) [18] displayed some similarity with an unknown protein predicted from a high expressed contig from *Erythrolamprus miliaris*. However, the areas of conservation were restricted to the signal peptide and to the *C*-terminal and thus it is not likely that the two putative proteins correspond to a common toxin (data not shown).

## 3. Conclusions 

It is now abundantly clear that the venoms produced among the colubrid rear-fanged snakes are homologous with the much better characterized venoms of the front-fanged snakes. As trophic adaptations that facilitate feeding, venoms vary in composition with several important factors, including phylogeny, and so it is to be expected that among the diverse colubrid lineages, novel compounds, and new functional variants of better-known venom proteins, will be encountered. Much progress toward understanding rear-fanged snake venom composition has been made in the last decade, but, as indicated above, we have barely begun to explore the diversity of advanced snakes that comprise the colubrids. Transcriptomic and genomic approaches will greatly facilitate this work, but it must be remembered that functional assays should also accompany analysis of any venom, because the common recurring motif in venom biochemistry is to make the most of a stable molecular scaffold, perhaps best exemplified by the varied pharmacologies of the three-finger toxin superfamily. These small, structurally conservative peptides have very similar crystal structures but affect systems as diverse as neurotransmission, the blood clot cascade, ion channel function, and salamander limb regeneration and courtship. As Dr. Jay Fox once said, in venoms “we find only what we are looking for”, and, to find truly novel toxins that will likely be present in some colubrid venoms, we will have to look beyond the “normal” families of venom proteins.

## 4. Materials and Methods 

### 4.1. Original Transcriptomic Data

#### 4.1.1. Animals

Three specimens of *Erythrolamprus miliaris* (one male and two females) five specimens of *Oxyrhopus guibei* (two males and three females) and two specimens of *Xenodon merremi* (both female) were provided by the Laboratory of Herpetology at the Instituto Butantan. These animals were collected from the wild by the local population, delivered at Instituto Butantan and kept in captivity for a short time (up to one month); all snakes were provided water *ad lib* but not fed. Manual extraction of the venom was performed 4 days prior to euthanizing the animals and dissecting out both Duvernoy’s venom glands, which were frozen in liquid nitrogen. All animal procedures were authorized by the Ethical Committee for Animal Research of Butantan Institute (protocols 164/2004 and 935/12, approved on 11 May 2004 and 1 June 2012, respectively), according to principles adopted by the Brazilian College of Animal Experimentation.

#### 4.1.2. RNA-Seq

*Erythrolamprus miliaris* and *Oxyrhopus guibei* transcriptomes were investigated using RNA-Seq, in a 454 pyrosequencing platform. Pairs of glands from each specimen were ground into a powder in liquid nitrogen and homogenized using a Polytron Tissue Homogenizer (Kinematica, Luzern, Switzerland). Total RNA was extracted with TRIZOL Reagent (Life Technologies, Thermo Fisher Scientific, Carlsbad, CA, USA) and mRNA was prepared using the Dynabeads mRNA DIRECT kit (Life Technologies, Thermo Fisher Scientific, Carlsbad, CA, USA). mRNA was quantified by the Quant-iT^TM^ RiboGreen RNA reagent and kit (Life Technologies, Thermo Fisher Scientific, Carlsbad, CA, USA). To obtain 500 ng of mRNA needed to prepare cDNA libraries for pyrosequencing with cDNA Synthesis System kit (Roche Diagnostics, Basel, Switzerland), we pooled mRNAs from individual specimens of each species. Emulsion PCR amplification and library sequencing were performed individually for each species, using a GS Junior 454 Sequencing System (Roche Diagnostics, Basel, Switzerland) according to the manufacturer’s protocols. The raw sequences were deposited in GenBank SRA with the accession numbers SRR3141951-SRR3141952 (*Erythrolamprus miliaris*) and SRR3141953 (*Oxyrhopus guibei*). 

The raw reads from each species were assembled with Newbler 2.7 (Roche Diagnostics, Basel, Switzerland), which first removes adaptors and contaminating ribosomal RNA sequences. The assembly parameters were set to: (i) a minimum overlap length of 50% of the read; and (ii) a minimum overlap identity of 98%, with all other parameters set as default. The resulting unigenes were deposited in the GenBank TSA repository with the accession numbers GEFK00000000.1 linked to Bioproject PRJNA310611 (*Erythrolamprus miliaris*) and GEFL00000000.1 linked to Bioproject PRJNA310661 (*Oxyrhopus guibei*). Unigene sequences were automatically annotated using Blast2Go [101] by performing a Blast search against the UniProt database with the algorithm BlastX to identify similar sequences. Toxin categories were manually assigned by comparing the unigenes to a compiled list of known snake toxins. Final manual curation of relevant unigene sequences was undertaken to improve the quality and the extension of the automatically assembled unigenes. The levels of expression of individual unigenes were calculated using the RNA-Seq function of CLC Genomics Workbench v8 (Qiagen, Hilden, Germany, 2015)) by mapping cleaned reads (without known contaminants and rRNAs) back to the unigenes and normalizing the count number by the length of the unigene using RPKM (reads per kilobase per million of mapped reads) formula [102].

#### 4.1.3. Expressed Sequence Tags (ESTs) Generation

*Xenodon merremi* transcriptome was investigated by means of EST generation, prior to the common use of NGS (next generation sequencing). The pairs of glands from *Xenodon merremi* specimens were ground into a powder in liquid nitrogen and homogenized using a Polytron Tissue Homogenizer (Kinematica, Luzern, Switzerland). Total RNA was extracted with TRIZOL reagent (Life Technologies, Thermo Fisher Scientific, Carlsbad, CA, USA) and mRNA was prepared using a column of oligo-dT cellulose (GE). To obtain 5 µg of mRNA needed to prepare cDNA libraries using the Superscript Plasmid System for cDNA Synthesis and Cloning (Life Technologies, Thermo Fisher Scientific, Carlsbad, CA, USA), we pooled mRNAs from the two specimens. The cDNA was ligated with the adaptors included in the kit, size selected into two ranges (250–600 bp and over 600 bp), directionally cloned into pSPORT-1 plasmids and transformed in *E. coli* DH5α electrocompetent cells. Plasmid DNA was isolated using alkaline lysis and sequenced on an ABI 3100 sequencer using the BigDye 3.1 kit (Applied Biosystems, Foster City, CA, USA) with a standard 5′ primer (M13R). The electropherogram files were analyzed in a semi-automatic way and then assembled in clusters of contiguous sequences using the CAP3 program [103] set for 98% or more of base identity in a high-quality region. The resulting unigenes were deposited in GenBank TSA repository with the accession number GETV00000000 linked to Bioproject PRJNA310192. The relative representation of each cluster was given by the number of ESTs used in its assembly, as described elsewhere [104].

### 4.2. Public Database Sequence Retrieval

Prototypical sequences of the different kinds of proteins known in the venoms of Viperidae, Elapidae, Atractaspididae and Colubridae snakes were compiled from GenBank and from our archives and used as in silico probes to a more extensive search for related protein sequences from Colubroidea snakes. Searches were performed using a stand-alone Blast tool of CLC Genomics Workbench v8 against a downloaded version of the GenBank nr database (December 2015), which includes non-redundant protein sequences from GenBank database and protein sequences from TSA (transcriptome shotgun assembly) repository. An initial e-value cutoff of lower than 10^−5^ was considered but the alignment of each sequence identified was individually evaluated to decide for the retrieving of a given protein. Whenever possible, the expression level of the transcript coding for each protein was examined from the bibliographical reference associated with the sequence to assign the “T” or “t” symbols on the summarizing tables, corresponding, respectively, to “high” (meaning highly expressed or higher expressed than in other tissues) or “low” (meaning lowly expressed or lower expressed than in other tissues) transcriptional level in the venom glands. Evidence for proteomic identification of proteins was also obtained from the literature.

### 4.3. Sequence Comparisons and Evolutionary Analyses

Protein sequences were incorporated into gene family sequence alignments containing toxin and non-toxin protein homologs and paralogs and representative outgroup sequences. Alignments were performed using MUSCLE or CLUSTALW tools of CLC Genomics Workbench v8 (Qiagen) and checked manually. Phylogenetic trees were generated by the Maximum Likelihood method, with WAG substitution model and bootstrapping 1000 replicates, using CLC Genomics Workbench.

## Figures and Tables

**Table 1:** Major snake venom components and their occurrences in colubrid species.

Species	Enzymatic				Non-Enzymatic							Reference
	LAAO	PLA2 (IA)	SVMP	SVSP	3FTx	CNP	CRISP	CTL	DEFEN	KUN-1	KUN-2	
*Boiga dendrophila*					B							[42,43]
*Boiga irregularis*			TPB		TPB	T	TP	T			t	[12,44,45]
*Borikenophis portoricensis*			B				BP					[46,47]
*Cerberus rynchops*			TP				TP	TP				[48]
*Coelognathus radiatus*					B							[49]
*Dispholidus typus*			xP		x		x					[50,51,52]
*Erythrolamprus miliaris*			T		t		T	T				This work; [50]
*Erythrolamprus poecilogyrus*			x		x		x	x				[50,51]
*Helicops angulatus*							BP					[53]
*Hypsiglena* sp.			TP		T	T	TP	TP			t	[13]
*Hypsiglena torquata*							P					[54]
*Leoiheterodon madagascarensis*					x		x	x				[50]
*Macropisthodon rudis*			t									[55]
*Opheodrys aestivus*					x		t	t			t	[9]
*Oxybelis fulgidus*					B							[56]
*Oxyrhopus guibei*			T			t	T	T			t	This work
*Phalotris mertensi*	TP		T	tP	t	t	t	T	TP	TP		[57]
*Pantherophis guttatus*			t		x		t	t			t	[9]
*Philodryas baroni*							P					[54]
*Philodryas chamissonis*			x	x		x	x	x				[58]
*Philodryas olfersii*			xTP	xTP		T	xTP	TP		x		[51,59]
*Philodryas patagoniensis*							P					[54]
*Pseudoferania polylepis*			x		x		x	x				[50]
*Rhabdophis tigrinus*						x	x	t				[50]
*Telescopus dhara*			x		x		x			x		[50,51]
*Thamnodynastes strigatus*			TP	t *	t		TP	TP	T			[60]
*Thrasops jacksonii*			x		x			x				[51]
*Trimorphodon biscutatus*		B			B		B					[54,61]
*Xenodon merremi*			T		T	T *	T	T				This work

Protein categories are: LAAO, l-amino acid oxidase; PLA2 (IA), phospholipase A_2_ (type IA); SVMP, snake venom metalloproteinase; SVSP, snake venom serine proteinase; 3FTx, three finger toxin; CNP, C-type natriuretic peptide; CRISP, cysteine rich secretory protein; CTL, C-type lectin, DEFEN, defensin (crotamine-like); KUN-1, Kunitz type protein (type 1); and KUN-2, Kunitz type protein (type 2). Types of evidence: T = Expressed in VG transcriptome at high level; t = Expressed in VG transcriptome at low (or uninformed) level; x = RT-PCR (non-quantitative); P = Detected in the proteome by MS/MS; and B = Protein purified and/or activity tested from the Duvernoy’s venom. The green color graduation represents the strength of the combination of evidence for each product, from light (less) to dark (more). Note: * = only 3′UTR detected.

**Table 2:** Minor snake venom components and their occurrences in colubrid species.

Species	Enzymatic							Non-Enzymatic										Reference
	5NUCLEO	AChE	DPP	FactV	FactX	HYAL	PDE	AVIT	bPLA2i	CVF	CYST	gPLA2i	KU-WA-FU	NGF *	OHA	VEGF-A **	WAP	
*Boiga dendrophila*																		[42,43]
*Boiga irregularis*		T				t	t		t	t	t	t				t	t	[12,44,45]
*Borikenophis portoricensis*		B					B											[46,47]
*Cerberus rynchops*																		[48]
*Coelognathus radiatus*																		[49]
*Dispholidus typus*																		[50,51,52]
*Erythrolamprus miliaris*		t	t			t				t		T				t	xt	This work; [50]
*Erythrolamprus poecilogyrus*																	x	[50,51]
*Helicops angulatus*																		[53]
*Hypsiglena* sp.			t										tP		t	t	t	[13]
*Hypsiglena torquata*																		[54]
*Leoiheterodon madagascarensis*																		[50]
*Macropisthodon rudis*							t											[55]
*Opheodrys aestivus*		t	t							t	x		t	x	t	t	t	[9]
*Oxybelis fulgidus*																		[56]
*Oxyrhopus guibei*		t	t							t		t				t	t	This work
*Phalotris mertensi*	tP	t							t		t	tP	t		tP	t	T	[57]
*Pantherophis guttatus*		t	t						***	t	t	***	t	x	t	t	t	[9]
*Philodryas baroni*																		[54]
*Philodryas chamissonis*																		[58]
*Philodryas olfersii*						t						T					x	[51,59]
*Philodryas patagoniensis*																		[54]
*Pseudoferania polylepis*																		[50]
*Rhabdophis tigrinus*																	x	[50]
*Telescopus dhara*																		[50,51]
*Thamnodynastes strigatus*		t				t												[60]
*Thrasops jacksonii*																	x	[51]
*Trimorphodon biscutatus*														x				[54,61]
*Xenodon merremi*			t *															This work

Protein categories are: 5NUCLEO, 5′nucleotidase; AChE, acetylcholinesterase; DPP, dipeptidyl peptidase; FactV, venom coagulation factor V; FactX, venom coagulation factor X; HYAL, hyaluronidase; PDE, phosphodiesterase; AVIT, AVIT protein; bPLA2i, beta type phospholipase A_2_ inhibitor; CVF, cobra venom factor; CYST, cystatins; gPLA2i, gamma type phospholipase A_2_ inhibitor; KU-WA-FU, ku-wap-fusin protein; NGF, nerve growth factor; OHA, ohanin (vesprin) protein; VEGF-A, vascular endothelial growth factor (type A); and WAP, waprin-like proteins. Types of evidence: T = Expressed in VG transcriptome at high level; t = Expressed in VG transcriptome at low (or uninformed) level; x = RT-PCR (non-quantitative); P = Detected in the proteome by MS/MS; and B = Protein purified and/or activity tested from the Duvernoy’s venom. The color gradation represents the strength of the combination of evidence for each product, from light (less) to dark (more). Note: * partial sequences from other colubrids were PCR amplified as part of a phylogenetic study [62]; ** no VEGF-F (svVEGF) detected in colubrids; *** cDNA and protein isolated from liver and serum of *P. quadrivirgata* and *P. climacophora*.

**Table 3:** Putative new snake venom components identified from colubrid species.

Species	Enzymatic				Non-Enzymatic				Reference
	svLIPA	PLA_2_ (IIE)	PLB	svMMP	EGFr	Lacta	LIPO	Vefico	
*Boiga dendrophila*									[42,43]
*Boiga irregularis*	t	t	t					t	[12,44,45]
*Borikenophis portoricensis*									[46,47]
*Cerberus rynchops*								TP	[48]
*Coelognathus radiatus*									[49]
*Dispholidus typus*		x					x		[50,51,52]
*Erythrolamprus miliaris*			t	T			T		This work; [50]
*Erythrolamprus poecilogyrus*				x					[50,51]
*Helicops angulatus*									[53]
*Hypsiglena* sp.								t	[13]
*Hypsiglena torquata*									[54]
*Leoiheterodon madagascarensis*		x							[50]
*Macropisthodon rudis*									[55]
*Opheodrys aestivus*	t	t						t	[9]
*Oxybelis fulgidus*									[56]
*Oxyrhopus guibei*	t	T	t	t		T	T	t	This work
*Phalotris mertensi*	TP		t	tP				t	[57]
*Pantherophis guttatus*	t	t	t						[9]
*Philodryas baroni*									[54]
*Philodryas chamissonis*									[58]
*Philodryas olfersii*	t		T	t				t	[51,59]
*Philodryas patagoniensis*									[54]
*Pseudoferania polylepis*								x	[50]
*Rhabdophis tigrinus*				TB			x		[50]
*Telescopus dhara*									[50,51]
*Thamnodynastes strigatus*				TP	T	TP			[60]
*Thrasops jacksonii*									[51]
*Trimorphodon biscutatus*							x		[54,61]
*Xenodon merremi*									This work

Protein categories are: LIPA, snake venom acid lipase; PLA2 (IIE), phospholipase A_2_ (type IIE); PLB, phospholipase B; svMMP, snake venom matrix metalloproteinase; EGFr, EGF repeats protein; Lacta, lactadherin-like protein; LIPO, lipocalin; and Vefico, veficolin (ficolin-like). Types of evidence: T = Expressed in VG transcriptome at high level; t = Expressed in VG transcriptome at low (or uninformed) level; x = RT-PCR (non-quantitative); P = Detected in the proteome by MS/MS; and B = Protein purified and/or activity tested from the Duvernoy’s venom. The color gradation represents the strength of the combination of evidence for each product, from light (less) to dark (more).

## Supplementary Materials

The following are available online at www.mdpi.com/2072-6651/8/8/230/s1, Table S1: Assembled contigs from the species investigated and their expression values.

## Abbreviations

3FTx, three finger toxin; 5NUCLEO, 5′nucleotidase; AChE, acetylcholinesterase; AVIT, AVIT protein; bPLA2i, beta type phospholipase A_2_ inhibitor; CDS, coding DNA sequence; CNP, C-type natriuretic peptide; CRISP, cysteine rich secretory protein; CTL, C-type lectin, DEFEN, defensin (crotamine-like); CVF, cobra venom factor; CYST, cystatins; DEF, defensin; DPP, dipeptidyl peptidase; EGFr, EGF repeats protein; ESTs, expressed sequence tags; FactV, venom coagulation factor V; FactX, venom coagulation factor X; gPLA_2_i, gamma type phospholipase A_2_ inhibitor; HYAL, hyaluronidase; KUN-1, kunitz type protein (type 1); KUN-2, kunitz type protein (type 2); KU-WA-FU, ku-wap-fusin protein; LAAO, l-amino acid oxidase; Lactha, lactadherin-like protein; LIPA, snake venom acid lipase; LIPO, lipocalin; NGF, nerve growth factor; OHA, ohanin (vesprin) protein; PDE, phosphodiesterase; PLA_2_ (IA), phospholipase A_2_ (type IA); PLA_2_ (IIE), phospholipase A_2_ (type IIE); PLB, phospholipase B; RPKM, reads per kilobase per million of mapped reads; svMMP, snake venom matrix metalloproteinase; SVMP, snake venom metalloproteinase; SVSP, snake venom serine proteinase; TSA, transcriptome shotgun assembly; Vefico, veficolin (ficolin-like); VEGF-A, vascular endothelial growth factor (type A); WAP, waprin-like proteins.
